# A comparison between voxel-based cortical thickness and voxel-based morphometry in normal aging

**DOI:** 10.1016/j.neuroimage.2009.06.043

**Published:** 2009-11-01

**Authors:** Chloe Hutton, Bogdan Draganski, John Ashburner, Nikolaus Weiskopf

**Affiliations:** a Wellcome Trust Centre for Neuroimaging, UCL Institute of Neurology, University College London, 12 Queen Square, London WC1N 3BG, UK; b Max Planck Institute for Human Cognitive and Brain Sciences, Leipzig, Germany

**Keywords:** Morphometry, VBM, Cortical thickness, Aging

## Abstract

The morphology of cortical grey matter is commonly assessed using T1-weighted MRI together with automated computerised methods such as voxel-based morphometry (VBM) and cortical thickness measures. In the presented study we investigate how grey matter changes identified using voxel-based cortical thickness (VBCT) measures compare with local grey matter volume changes identified using VBM. We use data from a healthy aging population to perform the comparison, focusing on brain regions where age-related changes have been observed in previous studies. Our results show that overall, in healthy aging, VBCT and VBM yield very consistent results but VBCT provides a more sensitive measure of age-associated decline in grey matter compared with VBM. Our findings suggest that while VBCT selectively investigates cortical thickness, VBM provides a mixed measure of grey matter including cortical surface area or cortical folding, as well as cortical thickness. We therefore propose that used together, these techniques can separate the underlying grey matter changes, highlighting the utility of combining these complementary methods.

## Introduction

The morphometric analysis of magnetic resonance images (MRI) of the brain has become a widely used approach to investigate neuroanatomical correlates of both normal brain development and neurological disorders. Most typically, changes in grey matter have been assessed using T1-weighted images while changes in white matter are more typically explored using diffusion tensor imaging (DTI) or diffusion weighted imaging (DWI) e.g. Smith et al. (2006). Some of the most commonly used methods for investigating cortical grey matter in T1-weighted images include volumetric comparisons of manually, semi-automatically or automatically delineated neuroanatomical regions of interest, whole-brain voxel-based comparisons of grey matter and cortical surface-based comparisons of cortical thickness.

A commonly used method for performing voxel-based comparisons of grey matter is known as voxel-based morphometry or VBM (Ashburner and Friston, 2000; Wright et al., 1995). This method generally relies on the MR images first being matched up in a common space, then, after correcting for intensity non-uniformities, voxels are classified as grey matter, white matter and cerebral spinal fluid (CSF). The value at each voxel in the resulting tissue segments can be thought of as representing the proportion of the corresponding tissue in that voxel. After smoothing the grey matter segments to create normally distributed fields of the quantity of grey matter, voxel-wise comparisons can be made over the whole brain in a regression model. Typically the initial matching process involves linear and non-linear warping of the images, which means that voxels need to be stretched and compressed to match up the different subjects. The voxel values can be modulated (scaled) to account for the regional stretching and compression so that the resulting value at each voxel can be considered as a measure of local absolute volume. In the cortex, the local grey matter volume is dependent on local cortical thickness and/or surface area. In healthy brain development, VBM studies have identified grey matter differences associated with normal aging, navigation, arithmetic, linguistic and musical learning abilities (Good et al., 2001; Maguire et al., 2000; Mechelli et al., 2004; Sluming et al., 2002). Further, longitudinal VBM studies have demonstrated training-induced structural changes in the adult human brain (Draganski et al., 2004; Draganski et al., 2006). VBM studies have also identified subtle grey matter differences associated with neurological disorders including diseases with known brain pathology, e.g. Alzheimers disease, Huntington disease (Good et al., 2002; Muhlau et al., 2007) or primary (idiopathic) disorders with the assumption of normal brain morphology, e.g. idiopathic cervical dystonia (Draganski et al., 2003), and chronic pain syndromes (May et al., 1999; Schmidt-Wilcke et al., 2005).

Estimation of cortical thickness based on T1-weighted images represents a viable methodological alternative to volumetric measurements for assessment of subtle cortical changes in the human brain. The initial image processing steps required to calculate cortical thickness typically involve segmentation of the images into grey matter, white matter and CSF, as with VBM. However, the subsequent steps may require substantially different processing in order to provide an absolute measure of thickness across the cortical surface. Typically cortical thickness measurements involve identification of the inner and outer cortical boundaries or surfaces. This may be achieved using image information and surface geometry to construct or fit a representation of the grey and white matter surfaces (Fischl and Dale, 2000; Jones et al., 2000; MacDonald et al., 2000; Miller et al., 2000; Zeng et al., 1999). The thickness at each point on the grey matter surface is then given by a distance measure between corresponding points on the two surfaces. Investigating surface-based cortical thickness measures across different subjects requires methods to match corresponding anatomical regions of the cortical surfaces. For example, the FreeSurfer software (http://surfer.nmr.mgh.harvard.edu/, Center for Biomedical Imaging, Charlestown, MA) which has been widely used for measuring cortical thickness in MRI, averages across subjects using non-rigid high-dimensional spherical averaging to align cortical folding patterns (Fischl et al., 1999). Other methods for measuring cortical thickness define the grey and white matter boundaries on the basis of voxel information and the thickness is based on the length of the trajectory from one boundary to another. A distinct difference between these methods and surface-based cortical thickness measures is that thickness is calculated for every volumetric point within the cortex rather than on the surface (Hutton et al., 2008; Jones et al., 2000; Yezzi and Prince, 2003). Differences in cortical thickness have been identified in normal aging (Salat et al., 2004), human intelligence (Choi et al., 2008), cognitive performance (Dickerson et al., 2008), and in neurological disorders including Gilles de la Tourette syndrome in children (Sowell et al., 2008) and Huntington disease (Rosas et al., 2008).

Studies using both VBM and surface-based cortical thickness measurements to analyse a single data set have reported differences in the results of the two methods e.g. Blankstein et al. (2009) and Voets et al. (2008). Differences have been attributed both to biology (Voets et al., 2008) and/or methodology (Blankstein et al., 2009). It should be noted that to make robust comparisons between volume- (e.g. VBM) and surface-based anatomical measures (e.g. surface-based cortical thickness), a system is required in which these representations can be integrated e.g. Makris et al. (2006).

In this study we compare our voxel-based cortical thickness measure, (VBCT) (Hutton et al., 2008), with the well-established VBM technique (Ashburner and Friston, 2000) in terms of their sensitivity to cortical grey matter changes. The methods are both voxel-based and use the same preprocessing, but measure different features of the grey matter. With VBCT, the one-dimensional scalar thickness of the cortex at each voxel location is measured. Whereas with VBM the quantity of tissue within a voxel is measured which is dependent on the local cortical surface area (and hence cortical folding) as well as the local cortical thickness. Given that both measures are voxel-based, the same spatial registration method can be used so that the comparison can be made conveniently in the same volumetric space. We use a volumetric spatial registration method (DARTEL) which has been demonstrated to provide improved anatomical precision (Ashburner, 2007; Bergouignan et al., 2009; Klein et al., 2009; Yassa and Stark, 2009). By using preprocessing and registration methods that are common to both VBCT and VBM, the sensitivity of our VBCT technique can be compared with that of the well-established VBM procedure while minimising any possibly confounding methodological differences. We also describe and evaluate the impact of technical advancements of the previously published original VBCT method (Hutton et al., 2008) which include improved extraction of the cortex and a method to adjust for the spatial transformations and smoothing applied to warp the VBCT maps into the reference space.

We perform the comparison between VBM and VBCT using a data set comprising healthy adult subjects with a wide range of ages. A significant number of neuroimaging studies have been performed using a variety of morphometric analysis methods to investigate cortical changes associated with aging. For example healthy aging has previously been studied using both cortical thickness measurements (Fjell et al., 2009; Salat et al., 2004), and VBM (Good et al., 2001; Ohnishi et al., 2001; Tisserand et al., 2002), as well as other methods to compare grey matter density (Sowell et al., 2003), and ROI measurements (Raz et al., 1997; Tisserand et al., 2002). Generally, variability exists in the published results which may arise from differences in the age range, the overall health of the group studied, in particular regarding unidentified vascular disease as well as differences in the methods used. However a recent large scale multi-centre study investigated age-related cortical thinning and reported a number of regions where consistent age effects were observed (Fjell et al., 2009). In our current study, our goal is not to use the two methods to identify cortical changes associated with aging as such. Instead, the goal is to use this well-established paradigm to compare the age-related grey matter changes identified using VBCT and VBM. In the comparison, we focus on grey matter volume and cortical thickness both globally and within specific brain regions where consistent age effects have been most commonly reported.

## Methods

### Subjects

Data from 48 healthy subjects, aged between 22 and 60 (mean age = 37.3, 20 females, no significant difference between the age range for males and females) were used in this study. The data were originally acquired as a control group for a study investigating movement disorders. Approval for the study was obtained from the local ethics committee.

### Data acquisition

Data were acquired on a 1.5 T Sonata whole body scanner (Siemens Medical Systems, Erlangen, Germany), using a whole body coil for transmission and an 8-channel phased-array head coil for reception. Whole-brain structural scans were acquired using a Modified Driven Equilibrium Fourier Transform (MDEFT) sequence (Ugurbil et al., 1993) with a FLASH-EPI hybrid readout with two acquisitions per excitation (Deichmann, 2006). This sequence provides whole-brain anatomical data with an isotropic spatial resolution of 1 mm^3^ in a total scan time of 8 min. For each subject, 176 sagittal partitions were acquired with an image matrix of 256 × 224 (Read × Phase). Two-fold oversampling was performed in the read direction (superior/inferior direction) to prevent aliasing. Other image acquisition parameters were TR/TE/TI = 20.66/8.42/640 ms, BW = 178 Hz/Px, α = 25°. The sequence employed echo time shifting, navigator echoes, asymmetric k-space sampling and optimized fat suppression (Howarth et al., 2006) to improve the image quality. Special RF excitation pulses were used to compensate for B1 inhomogeneities of the transmit coil in superior/inferior (Deichmann et al., 2000) and anterior/posterior (Deichmann et al., 2002) directions.

### Data processing

#### Segmentation

**Fig. 1:**
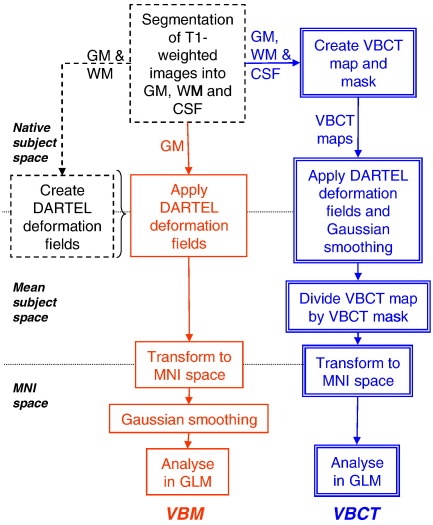
Overview of data processing steps used for the VBM and VBCT analysis. (See Methods section for details). VBM steps are indicated by a solid line (red), VBCT steps are indicated by a double line (blue) and steps common to both are indicated by a dotted line (black).

The data processing steps are illustrated in Fig. 1. Before preprocessing, all of the images were checked for artefacts and manually aligned so that the origin of the coordinate system was located at the anterior commissure. Using the unified segmentation procedure (Ashburner and Friston, 2005) implemented in SPM5 (http://www.fil.ion.ucl.ac.uk/spm), the images were segmented into grey matter (GM), white matter (WM) and cerebrospinal fluid (CSF). For each subject, this resulted in a set of 3 images in the same space as the original T1-weighted image, in which each voxel was assigned a probability of it being GM, WM and CSF respectively.

#### Creation of VBCT maps

A voxel-based cortical thickness (VBCT) map was created for each subject using the GM, WM and CSF segments created in the previous step. This method automatically extracts the cortical GM boundaries from T1-weighted images and estimates a value for the cortical thickness at each voxel. Some specific details about the method are given in the following paragraph but have been described in full detail previously (Hutton et al., 2008). Two modifications aimed at improving the extraction of the cortex were made for this study. The first development involved transforming a labelled brain atlas into the space of each individual subject to identify and exclude sub-cortical regions from the resulting estimation of VBCT. A brain atlas containing 71 segmented structures provided with the IBASPM toolbox (available at http://www.fil.ion.ucl.ac.uk/spm/ext/#IBASPM) (Aleman-Gomez et al., 2006) was used for this purpose. The second modification used a skeletonized representation of the binarised CSF segment to improve the delineation of deep sulci in the brain. Skeletonization is a process that erodes the voxels belonging to an object in a binary image resulting in a skeletal remnant that largely preserves the extent and connectivity of the original region (Jain, 1989). Prior to the calculation of thickness, the original CSF segments were updated with voxels from the skeletonized version of the CSF to better delineate the GM-CSF boundary, especially in small CSF spaces.

The initial step for the computation of the VBCT maps involved sub-sampling the input tissue segments from 1 mm to 0.5 mm using trilinear interpolation to allow smaller structures, particularly narrow CSF spaces, to be better resolved. An initial estimate of the GM/WM and GM/CSF boundaries was calculated from the input tissue probability maps and the transformed labelled brain atlas. From the initial estimate of the GM/WM boundary, layers of one voxel thick were successively added to surround the WM. By computing the thickness for each layer and comparing it to the expected thickness it was possible to identify voxels where the grey matter from different sides of a sulci was in contact (i.e. sulcal voxels). Once all the GM had been processed in this way, the final thickness was calculated by first assigning arbitrary boundary values to the GM/WM and GM/CSF boundaries and solving Laplace's equation for all voxels between them (Jones et al., 2000). The resulting scalar field makes a smooth transition from one boundary to the other so that the thickness between them can be calculated by integrating along unique trajectories connecting the two boundaries. The length of the trajectory through voxels identified as sulcal voxels was set to be half of the total possible trajectory through the voxel. The integration was performed using an integration step-size of half the sub-sampled voxel size, i.e. 0.25 mm with trilinear interpolaton. The resulting VBCT maps were saved with the native resolution and space of the original input images (i.e. 1 mm^3^). The resulting VBCT maps contained cortical thickness values within voxels identified as cortical grey matter and zeros outside the cortex.

#### Registration between subjects

DARTEL (Ashburner, 2007), an algorithm for diffeomorphic image registration, implemented as a toolbox for SPM5, was used to optimally warp the GM segments and the VBCT maps into a new reference space representing an average of all the subjects. The first step of the DARTEL procedure used GM and WM segments to create a set of group specific templates (Ashburner and Friston, 2009) and the deformation fields required to warp the data from each subject to the new reference space. For the VBM analysis, the next step used each subject specific deformation field to warp the corresponding GM segment into the new reference space, resampling it at 1.5 mm isotropic voxels using trilinear interpolation then the warped GM segments were affine transformed into MNI space. The GM probability values were scaled by the Jacobian determinants of the deformations to account for the local compression and stretching that occurs as a consequence of the warping and affine transformation. This process has been referred to as “modulation” (Good et al., 2001) and is based on the change of variables theorem (Boas, 1983). Finally, the GM probability values were smoothed using a 6 mm FWHM Gaussian kernel. The DARTEL procedure is a relatively recent alternative to previous spatial normalisation methods, e.g. Ashburner and Friston (1999, 2005), providing improved anatomical precision (Bergouignan et al., 2009; Klein et al., 2009; Yassa and Stark, 2009). A number of studies have demonstrated the accuracy of this method. In the largest evaluation of non-linear deformation algorithms to date (14 in total), DARTEL was shown to be as accurate as the other methods studied (Klein et al., 2009).

The warping and smoothing steps which were applied to the GM segments for the VBM analysis were modified for the VBCT maps. This was necessary because different features of the grey matter are measured with the local GM volume compared with the VBCT. The VBCT value at each voxel should be a fixed measure of the underlying anatomy and should not change even if the spatial registration procedures (i.e. DARTEL warping) change the voxel geometry. In contrast, the local GM volume is a measure of the quantity of tissue within a voxel which should change if the voxel geometry changes. When applying smoothing to the VBCT values, if the smoothing kernel is slightly larger than the underlying structure, the effect of Gaussian smoothing is to slightly reduce the original VBCT values. Furthermore, when Gaussian smoothing is applied to the warped VBCT maps, it is effectively being performed in the new reference space rather than the native space of each subject. To compensate for each of these effects, a modified warping and weighted smoothing procedure was implemented. The first step used each subject specific deformation field to warp the corresponding VBCT map into the new reference space. The warped images were resampled at 1.5 mm isotropic voxels using trilinear interpolation, rescaled by the Jacobian determinant of the deformations (i.e. modulation) and smoothed with a 6 mm Gaussian kernel. The same warps, modulation and smoothing were also applied to a binary mask of each corresponding original VBCT map. The processed VBCT map was then divided by the corresponding processed mask. This warping and weighted smoothing procedure effectively projects the Gaussian smoothing kernel which is applied in the DARTEL warped space into the native space of the subject while preserving the cortical thickness value over a region the size of the smoothing kernel. A similar approach to the latter was used to improve the results of a voxel-based analysis study of diffusion tensor imaging (Lee et al., 2009). Finally the warped, smoothed VBCT maps were affine transformed into MNI space. This involved changing the spatial transformation information only so that the thickness map values themselves were not affected.

### Comparison between VBCT and VBM

#### Global effects of age

The total GM volume (TGMV) was calculated for each subject by summing together the voxel values of the original grey matter tissue segments, including sub-cortical structures and the cerebellum. The mean cortical thickness (MCT) was calculated for each subject by summing together all of the thickness values within a subject specific grey matter mask and dividing by the number of voxels within the mask. The global effects of age on the TGMV and MCT were independently investigated using a multiple linear regression model which included age and gender. The global effects of age on TGMV and MCT were also investigated for males and females separately. *F*-tests were used to assess the significance of the regression models and *t*-tests were used to assess differences between males and females. A *P*-value of *P* < 0.05 (after a Bonferroni correction for all the tests performed) was considered to be significant.

#### Voxel-wise effects of age

Regional effects of age were assessed by performing voxel-wise multiple linear regression on the smoothed warped GM segments and VBCT maps independently using the General Linear Model framework implemented in SPM5. A mask of the grey matter was created by performing a logical ‘OR’ over all of the warped VBCT maps. This cortical GM mask was used in both the GM and VBCT analyses to ensure that the same voxels were included and the search volume was the same. The same regression model was used for both the GM segments and the VBCT maps. The model included age, gender and total intracranial volume (TIV) to account for any confounding effects of the overall brain size caused by gender or body size as well as a constant to model the mean. The TIV was calculated for each subject by summing together the total tissue probability values of the GM, WM and CSF probability maps resulting from the initial segmentation of the original images.

Voxel-wise *t*-tests were used to detect changes in local GM volume (GMV) and VBCT with age. Voxel-wise *F*-tests were used to compare the full regression model that included age, gender and TIV with a reduced model including only age and gender. The *F*-tests were performed to indicate how much of the variance in the GMV or VBCT data could be explained by the TIV over and above the variance explained by age and gender. For the voxel-wise tests, *P*-values were estimated on the basis of a family-wise error (FWE) correction for multiple comparisons over the cortical GM mask and considered to be significant for *P* < 0.05 (FWE corrected). Spatial maps of the standard error were generated for the GMV and VBCT data by calculating the square root of the sum of the squares of the difference between the full fitted model and the data divided by the number of degrees of freedom.

Spatial maps of age-related effects were projected onto a surface rendering of a middle layer of grey matter from a single subject brain which had been transformed into average subject space using DARTEL then into MNI space as described before. Maps of the model coefficients representing the mean and decrease with age, as well as the associated maps of *T*-scores and standard error were generated for the GMV and VBCT. The coefficients representing the mean and decrease in GMV with age were scaled by the resampled voxel volume to give maps in units of mm^3^. The coefficients representing the change in VBCT were in units of mm. Since the sample size used here (48 subjects) is smaller than typically used in aging studies e.g. Fjell et al. (2009), Good et al. (2001), Salat et al. (2004), and Sowell et al. (2003), the maps of *T*-scores are shown thresholded at a descriptive *P*-value of 0.05 to illustrate regional trends in the data.

##### ROI analyses

A set of regions of interest (ROIs) were defined using the IBASPM toolbox and included the left and right middle and superior frontal areas. These regions were selected because they have been commonly reported to show age-associated changes in the cortex (Fjell et al., 2009; Good et al., 2001; Salat et al., 2004; Sowell et al., 2003; Tisserand et al., 2002). Since the IBASPM toolbox is defined in MNI space, the spatial alignment of the ROIs was considered to have the same high accuracy as the DARTEL registration. For each ROI, the mean of the voxel-wise coefficients representing decrease with age and the median *T*-score for the GMV and VBCT data were extracted. The smoothed warped GMV and VBCT values were also extracted and plotted (mean and standard deviation) against age. The standard deviation was not corrected for spatial correlations within the ROI since it could be assumed that these were the same in the GMV and VBCT data. A measure of signal-to-noise ratio (SNR) was calculated for each method, each ROI and each subject. The SNR was calculated by dividing the mean of the data in each ROI by the standard deviation of the “noise”. The noise was calculated by subtracting the modelled effects from the data at each voxel in the ROI. A paired two-sided Wilcoxon signed rank test was used to determine whether the SNR was significantly greater in VBCT or VBM for each ROI. A *P*-value of *P* < 0.05 was considered to be significant after a Bonferroni correction for all the ROIs tested (i.e. 4).

### Evaluation of methodological advancements of VBCT measurements

The impact of the presented technical advancements on the VBCT method was evaluated using the regression model and pre-defined ROIs described above. Smoothed warped VBCT maps were generated using the previously published method (i.e. without the improved cortical extraction; Hutton et al., 2008), with an earlier method for transforming all the data into a common reference space (Ashburner and Friston, 2005) and with standard Gaussian smoothing of FWHM = 6 mm. The data were analysed for voxel-wise effects of aging as described above for the current VBCT method. For each ROI, the mean of the voxel-wise coefficients representing decrease with age and the median *T*-score for the GMV and VBCT data were extracted. The smoothed warped VBCT values were extracted for the old and the new VBCT methods and the SNR measure described above was calculated for each set of data, each ROI and each subject. A pair-wise comparison between the old and new VBCT methods was performed in each ROI using a paired two-sided Wilcoxon signed rank test to determine whether the SNR was significantly greater in the VBCT data processed by the new or the old method. A *P*-value of *P* < 0.05 was considered to be significant after a Bonferroni correction for all the ROIs tested (i.e. 4).

## Results

### Comparison between VBCT and VBM

#### Global effects of age

**Fig. 2:**
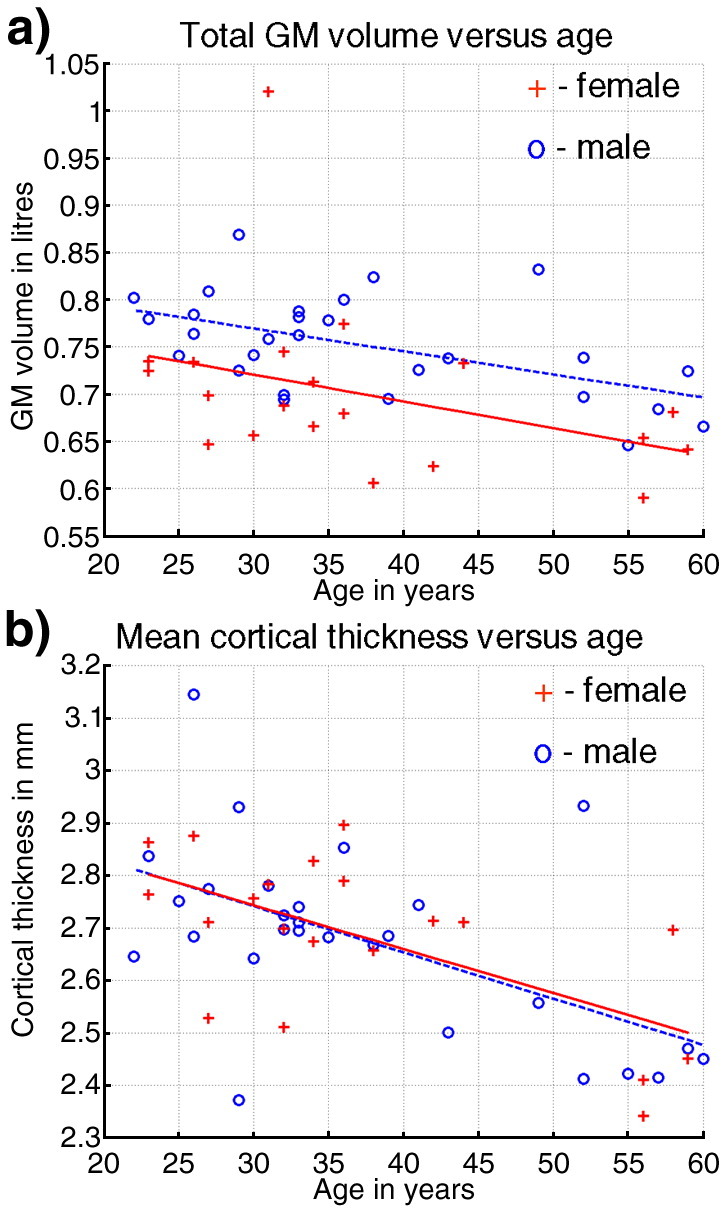
Global effects of age on the brain. (a) Total GM volume in litres versus age. (b) Mean cortical thickness in mm versus age. Females = ‘+’, males = ‘o’. The solid and dotted lines shows the linear regression of age on the data for females and males respectively.

The total GM volume in litres (TGMV) versus age in years is shown in Fig. 2a. All *P*-values were Bonferroni corrected. For males and females together, there was a significant global decline in TGMV with age (*R*^2^ = 0.28, the linear coefficient, B1 = − 0.0026 l per year, *F*(1,45) = 8.70, *P* < 0.01). For males (circles), the decline with age was also significant (dotted line, *R*^2^ = 0.28, B1 = − 0.0028 l per year, *F*(1,24) = 9.98, *P* < 0.05). The results for the females (crosses) had a similar trend to the males but did not reach significance (solid line, *R*^2^ = 0.14, B1 = − 0.0024 l per year, *F*(1,16) = 2.82, *P* = 0.4). There was a difference in the mean TGMVs for males and females, which almost reached significance (*P* = 0.051) but no significant difference in decline with age. The mean cortical thickness in mm (MCT) versus age in years is shown in Fig. 2b. For males and females together, there was a significant global decline in MCT with age, (*R*^2^ = 0.34, B1 = − 0.0086 mm per year, *F*(1,45) = 11.65, *P* < 0.001). For males (circles), the decline with age was significant (dotted line*, R*^2^ = 0.39, B1 = − 0.0088 mm per year, *F*(1,24) = 12.07, *P* < 0.05) and also for the females (crosses and solid line, *R*^2^ = 0.34, B1 = − 0.0086 mm per year, *F*(1,17) = 11.65, *P* < 0.01). No differences were observed between males and females for the MCT.

#### Voxel-wise effects of age

The voxel-wise multiple linear regression of age on the GMV and the VBCT maps identified several brain regions which have been reported previously in neuroimaging studies of healthy aging. All of the regions that are reported here as being significant survived a statistical threshold of *P* < 0.05 (FWE corrected). Regions where significant age-related effects were observed in both the GMV and the VBCT, were the left and right insula, left superior temporal and left medial frontal gyrus. Significant age-related decreases in GMV were also observed in inferior frontal, middle temporal, lateral occipital temporal and postcentral gyrus all on the left side only. Decreases in VBCT were also observed in these regions but at a less conservative threshold of *P* < 0.001 (T(44) = 3.286). Significant age-related decreases in VBCT were observed bilaterally in middle and superior frontal, superior temporal, precentral gyrus and on the right side only in postcentral gyrus. Decreases in GMV were also observed in these regions but at a less conservative threshold of *P* < 0.001 (T(44) = 3.286). Overall, at the less conservative threshold of *P* < 0.001 (T(44) = 3.286), more widespread age-related decreases in GMV and VBCT were observed in prefrontal, temporal and orbitofrontal regions, insula, cingulate, and precentral gyri. At this threshold, the patterns of age-related changes in the GMV and the VBCT were more similar.

**Fig. 3:**
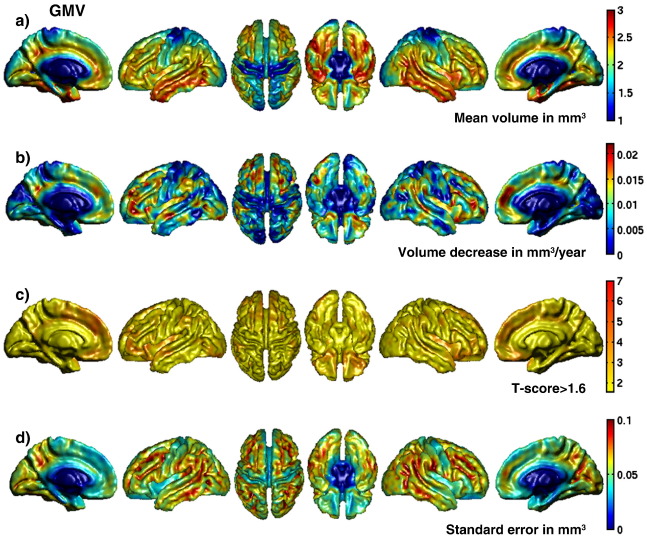
Regional effects of age on local GM volume rendered onto surface of spatially normalised grey matter. (a) Mean local GM volume in mm^3^. (b) Decrease in local GM volume in mm^3^ per year. (c) Voxel-wise *T*-scores for decrease in local GM volume with age, (*T*-score > 1.6, corresponding to a descriptive *P*-value threshold of *P* < 0.05). (d) Voxel-wise standard error (in mm^3^) calculated from the square root of the sum of the squares of the difference between the full fitted model and the GM volume data divided by the number of degrees of freedom. Although sub-cortical regions are visible in the medial and inferior views these were not included in the analyses and have therefore been set to the minimum value on the colour scale.

**Fig. 4:**
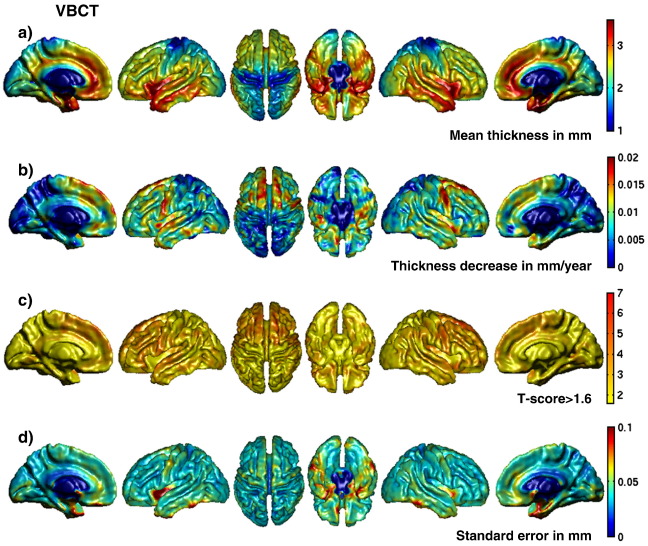
Regional effects of age on VBCT rendered onto surface of spatially normalised grey matter. (a) Mean VBCT in mm. (b) Decrease in VBCT in mm per year. (c) Voxel-wise *T*-scores for decrease in VBCT with age, (*T*-score > 1.6, corresponding to a descriptive *P*-value threshold of *P* < 0.05). (d) Voxel-wise standard error (in mm) calculated from the square root of the sum of the squares of the difference between the full fitted model and the VBCT data divided by the number of degrees of freedom. Although sub-cortical regions are visible in the medial and inferior views these were not included in the analyses and have therefore been set to the minimum value on the colour scale.

Figs. 3a and b show the surface-rendered maps of coefficients representing the mean GMV in mm^3^ and the decrease in GMV with age in mm^3^ per year. Figs. 4a and b show the surface-rendered maps of coefficients representing the mean VBCT in mm and the decrease in VBCT with age in mm per year. From Figs. 3a and 4a, it can be seen that the overall spatial pattern of GMV and VBCT are very similar. From Fig. 3b, GMV decreases of up to 0.02 mm^3^ per year can be observed in localised prefrontal, temporal and cingulate regions and from Fig. 4b, VBCT decreases of up to 0.02 mm per year can be observed in more widespread prefrontal, orbitofrontal, temporal areas, precentral gyral and cingulate regions. The maps of *T*-scores corresponding to the decrease with age in GMV and VBCT are shown in Figs. 3c and 4c respectively. To facilitate detailed exploration, these have been thresholded at *T* > 1.6 corresponding to a descriptive *P*-value of *P* < 0.05 and scaled equivalently up to *T* = 7 (*P* < 0.0001). By comparing Figs. 3c and 4c, it can be observed that overall the *T*-scores for the decrease in VBCT with age are greater and more spatially widespread compared with decreases in GMV with age. Figs. 3d and 4d show the surface-rendered standard error maps for GMV and VBCT respectively which provide a spatial pattern of how well the regression model fits each set of data. From Fig. 4d, it appears that the fit of the regression model is worse in voxels located within deep sulci for the GMV data.

The voxel-wise *F*-tests used to indicate how much of the variance in the GMV or VBCT data could be explained by the TIV, demonstrated that for the GMV analysis, variance in voxels covering much of the cortex could be significantly explained by the TIV (*P* < 0.05, FWE corrected, results not shown). In contrast, for the VBCT analysis no regional changes were significantly correlated with the TIV regressor.

#### ROI analyses

**Fig. 5:**
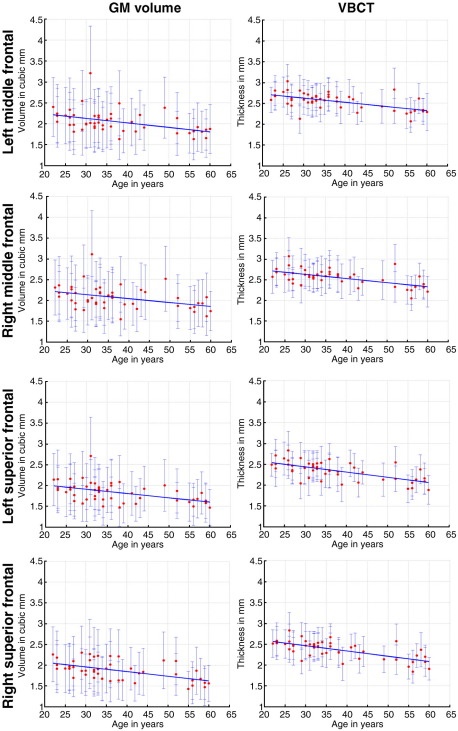
Effect of age on local GM volume (in mm^3^, left column) and VBCT (in mm, right column) in left and right middle frontal and left and right superior frontal areas. The dots show the mean and the errorbars show the standard deviation (not corrected for spatial correlations) of the local GM or VBCT within the ROI for each subject. The solid lines show the linear regression of age on the data within the cluster.

Fig. 5 and Table 1 show the data and statistics from selected ROIs which have been associated with age-related decreases in GM; right and left middle and superior frontal regions. In Fig. 5 the dots represent the mean GMV or VBCT value in each ROI and the error bars are the standard deviations (not corrected for spatial correlations) of the data within the ROI. Although the GMV and VBCT units on the *y*-axes are not the same, the plots have been scaled equivalently for comparison of the error bars. From Fig. 5 (solid lines) and Table 1 (B1) it can be seen that the linear regression of age on the data are of a similar order for all regions, although it must be noted that for GMV the regression is in mm^3^/year whereas for VBCT it is mm/year. From Table 1 it can also be seen that for all ROIs the median *T*-score is larger for the VBCT compared with the VBM data. Furthermore, for each ROI, the mean of the estimated SNR over all of the subjects is greater for the VBCT compared with the VBM data. This is also apparent in the relative size of the error bars in Fig. 5. The paired two-sided Wilcoxon signed rank test demonstrated that for all ROIs, the SNR was significantly greater in the VBCT data than the VBM data, (*P* < 0.05, Bonferroni corrected).

### Evaluation of methodological advancements of VBCT measurements

The comparisons between age-associated effects for the new VBCT and old VBCT data in selected ROIs are given in Table 1. The linear regression of age on the data (B1) are of a similar order for all regions, the median *T*-score and the mean of the estimated SNR over all of the subjects is greater for the new VBCT compared with the old VBCT data. The paired two-sided Wilcoxon signed rank demonstrated that for all ROIs, the SNR was significantly greater in the new VBCT data than old VBCT data, (*P* < 0.05, Bonferroni corrected).

## Discussion

This study compares a commonly used technique for assessing voxel-based morphometric differences in the brain (VBM; Ashburner et al., 2003) with an optimized method to calculate voxel-based cortical thickness (VBCT). The aim of the study was to identify how results from the two methods would differ by using them to assess grey matter changes in a healthy aging population. In addition, technical advancements of our previously published VBCT (Hutton et al., 2008) method have been described and evaluated. The goal of these modifications has been to improve the sensitivity and specificity of the extracted measure of cortical thickness and voxel-wise group analyses performed on this measure. The improvement was demonstrated by comparing the results of voxel-wise analyses performed on data processed using the previously published VBCT method with the current one in specific ROIs where aging effects have been shown to be consistent (Fjell et al., 2009). The linear coefficients representing decrease in cortical thickness with age were similar but median *T*-scores were consistently larger and SNR was significantly greater for the new VBCT method compared to the original method.

The spatial distribution over the brain of the voxel-wise mean of grey matter volume (GMV) and VBCT were very similar when the results were displayed at a relatively liberal descriptive *P*-value of *P* < 0.001. The overall mean and standard deviation for the VBCT over all subjects studied was 2.7 ± 0.2 mm. This value falls within the range of thickness values reported by previous studies of cortical thickness using both histological and MRI-based measures e.g. Fischl and Dale (2000), Kabani et al. (2001) and Rosas et al. (2002).

The spatial distribution of decline with age of GMV and VBCT were also very similar when the results were displayed at a relatively liberal descriptive *P*-value of *P* < 0.001. The main regions of cortical grey matter decline with age included prefrontal, orbitofrontal and temporal regions, insula, cingulate and precentral sulcus which are regions that have been reported in the literature (Fjell et al., 2009; Good et al., 2001; Salat et al., 2004; Sowell et al., 2003; Tisserand et al., 2002). At a *P*-value of *P* < 0.05 (FWE corrected), decreases in left and right insula, left superior temporal and left medial frontal gyrus could be observed in both the GMV and the VBCT results. For the VBCT, significant age-related decreases were also observed bilaterally in middle and superior frontal gyri and superior temporal gyri which are regions where consistent aging effects have been reported across multiple samples (Fjell et al., 2009). Age-related GMV decreases were also observed in these regions but at a descriptive *P*-value of *P* < 0.001. A possible reason for the difference in sensitivity between the GMV and the VBCT results is that although cortical thickness changes can be detected in the GMV measure, because GMV is also dependent on surface area and therefore possibly cortical folding, it is less sensitive to specific changes in thickness compared with VBCT. The decline in thickness for the specific ROIs analysed in this study was of the order of 0.01 mm per year (i.e. approximately 0.4% per year). This is greater than the results reported in Salat et al. (2004) which could be explained by the different age ranges between the two studies (75 versus 38 years in this study) and/or the number of subjects studied (106 versus 48 in this study). Furthermore, different rates of estimated age-related decline in cortical thickness have also been reported when multiple samples were analysed (Fjell et al., 2009).

When investigating the global effect of aging on the brain both the total grey matter volume (TGMV) and the mean cortical thickness (MCT) yielded significant linear decreases, with the correlation coefficient and statistical significance being slightly higher for the MCT compared to the TGMV. These results showed that although both TGMV and MCT change with age, TGMV is able to separate males and females whereas the MCT is not. This finding reflects the fact that overall brain size is larger for males compared to females and that cortical thickness changes very little with overall brain size. This is in agreement with the literature which suggests enlargement of the cortex can be attributed to an increase in cortical surface area rather than thickness e.g. Rakic (1995). Furthermore, these results suggest that by using VBCT together with VBM, underlying grey matter changes can be separated and better understood.

Comparing the regional effects of age between VBCT and GMV, indicated that for the VBCT data, the linear regression of age resulted in overall higher *T*-scores. This trend was also reflected when comparing the VBCT with GMV results in specific ROIs where consistent aging effects have been shown (Fjell et al., 2009). Larger SNRs and median *T*-scores were consistently observed in the VBCT data compared with the GMV data. The statistical comparison between the SNR for the VBCT and GMV data demonstrated a significantly better SNR for the VBCT data. Possible explanations for these differences may be that GMV is confounded by local cortical surface area or folding and/or that the GMV estimate is more sensitive to image noise. The former idea is supported by the spatial maps of standard error which indicate that the fit of the regression model is worse in voxels located within deep sulci for the GMV data. These are voxels where one may expect a larger local surface area due to a higher degree of cortical folding which is not being explained by the model. Furthermore, the results of the *F*-test indicated that GMV variance over large regions of the brain could be explained by the total intracranial volume (TIV) whereas variance in the VBCT was not explained by the TIV. This regressor is included to account for overall differences in brain size and is often used in VBM studies. A similar rationale does not exist for including the mean cortical thickness as a confound since we assume, as suggested by Rakic (1995), that local cortical surface area rather than cortical thickness varies with total intracranial volume. Therefore, age-related changes in local grey matter volume may also be confounded by total intracranial volume. From these results, one can expect that cortical thickness can be used to selectively investigate atrophy while VBM provides a mixed measure of cortical grey matter including cortical surface area or cortical folding, as well as cortical thickness. Consequently, in certain situations, VBCT is expected to be more sensitive than GMV/VBM, for example, if there is a prior hypothesis that grey matter changes are mainly due to changes in cortical thickness and also if there is any correlation between the effect of interest and the total brain volume. However, when used together, these techniques can separate the underlying grey matter changes, highlighting the utility of combining these complementary methods.

An important consideration for this study is that the MRI acquisition sequence used was a fast hybrid MDEFT sequence acquired at 1.5 T (Deichmann, 2006), with a relatively short acquisition time of 8 min and therefore lower SNR compared with long T1-weighted sequences often used in VBM studies (Deichmann et al., 2004). Furthermore, the number of subjects used in this study was 48 subjects over an age range of 38 years which is a smaller sample than is typically used in aging studies, e.g. Good et al. (2001), Salat et al. (2004) and Sowell et al. (2003). It is therefore possible that the lower statistical significance of the results reported for the GMV data is related to having less power in the data to start with and that the more constrained VBCT measure compensates for this somewhat. This suggests that VBCT is particularly useful for smaller datasets with lower SNR.

## Conclusion

The results of this study show that VBM and VBCT yield overall consistent results when investigating healthy aging. However, based on the data presented here, the VBCT method provides a more sensitive measure of age-associated decline in grey matter compared with the GMV measure typically used in VBM studies. We conclude that VBM and VBCT should be considered as complementary approaches: VBCT specifically measuring the cortical thickness and VBM being additionally sensitive to local surface area and cortical folding.

## Figures and Tables

**Table 1:** Table of ROI values corresponding to Fig. 5 for local GM (GMV), (new) VBCT and (old) VBCT_OLD_ results.

	B1	Median (*T*-score)	Mean (SNR)
*Left middle frontal area*			
GMV	− 0.011	2.01	5.71
VBCT	− 0.010	3.02	12.02
VBCT_OLD_	− 0.011	2.73	10.03

*Right middle frontal area*			
GMV	− 0.009	1.86	5.67
VBCT	− 0.010	3.02	12.42
VBCT_OLD_	− 0.012	2.92	10.58

*Left superior frontal*			
GMV	− 0.010	2.30	5.97
VBCT	− 0.013	3.50	10.23
VBCT_OLD_	− 0.015	3.30	8.47

*Right superior frontal*			
GMV	− 0.011	2.52	5.74
VBCT	− 0.013	3.62	10.52
VBCT_OLD_	− 0.015	3.36	8.41

B1 is the linear coefficient representing decrease with age (in mm^3^/year for GMV and mm/year for VBCT and VBCT_OLD_). Median (*T*-score) is calculated over all *T*-scores in each ROI. Mean (SNR) is calculated over subjects. For each subject, the SNR is calculated by dividing the mean of the data by a measure of noise in the ROI. The noise is calculated by subtracting the modelled effects from the data and calculating the standard deviation of the result in each ROI.
